# Is the concomitant use of clopidogrel and Proton Pump Inhibitors still associated with increased adverse cardiovascular outcomes following coronary angioplasty?: a systematic review and meta-analysis of recently published studies (2012 – 2016)

**DOI:** 10.1186/s12872-016-0453-6

**Published:** 2017-01-05

**Authors:** Pravesh Kumar Bundhun, Abhishek Rishikesh Teeluck, Akash Bhurtu, Wei-Qiang Huang

**Affiliations:** 1 Institute of Cardiovascular Diseases, the First Affiliated Hospital of Guangxi Medical University, Nanning, Guangxi 530021 People’s Republic of China; 2 Guangxi Medical University, Nanning, Guangxi 530027 People’s Republic of China

**Keywords:** Proton pump inhibitors, Clopidogrel, Percutaneous coronary intervention, Major adverse cardiac events

## Abstract

**Background:**

Controversies were previously observed with the concomitant use of clopidogrel and Proton Pump Inhibitors (PPIs), especially omeprazole, following coronary angioplasty. Even though several studies showed no interaction between clopidogrel and PPIs, questions have been raised about the decrease in antiplatelet effects of clopidogrel with PPIs. A previously published meta-analysis showed concomitant use of clopidogrel and PPIs to be associated with higher adverse cardiovascular outcomes. However, data which were used were extracted from studies published before the year 2012. Whether these controversies still exist in this new era is not clear. Therefore, we aim to show if the concomitant use of clopidogrel and PPIs is still associated with higher adverse outcomes following Percutaneous Coronary Intervention (PCI) using data obtained from recently published studies (2012 to 2016).

**Methods:**

Electronic databases were searched for recent publications (2012–2016) comparing (clopidogrel plus PPIs) versus clopidogrel alone following PCI. Adverse cardiovascular outcomes were considered as the clinical endpoints. Odds Ratios (OR) with 95% Confidence Intervals (CI) were used as the statistical parameters and the pooled analyses were performed with RevMan 5.3 software.

**Results:**

Eleven studies with a total number of 84,729 patients (29,235 patients from the PPIs group versus 55,494 patients from the non-PPIs group) were included. Results of this analysis showed that short term mortality and Target Vessel Revascularization (TVR) significantly favored the non-PPIs group with OR: 1.55; 95% CI: 1.43–1.68, *P* < 0.00001 and OR: 1.26; 95% CI: 1.06–1.49, *P* = 0.009 respectively. Long-term Major Adverse Cardiac Events (MACEs), Myocardial Infarction (MI), Stent Thrombosis (ST) and TVR significantly favored patients who did not use PPIs with OR: 1.37; 95% CI: 1.23–1.53, *P* < 0.00001, OR: 1.41; 95% CI: 1.26–1.57, *P* < 0.00001 and OR: 1.38; 95% CI: 1.13–1.70, *P* = 0.002 and OR: 1.28; 95% CI: 1.01–1.61, *P* = 0.04 respectively. However, the result for long term mortality was not statistically significant.

**Conclusion:**

The combined use of clopidogrel with PPIs is still associated with significantly higher adverse cardiovascular events such as MACEs, ST and MI following PCI supporting results of the previously published meta-analysis. However, long-term mortality is not statistically significant warranting further analysis with randomized patients.

## Background

Controversies still exist with the concomitant use of clopidogrel, one of the components of the Dual Anti-Platelet Therapy (DAPT) with Proton Pump Inhibitors (PPIs), especially omeprazole following Percutaneous Coronary Intervention (PCI). Even if the American College of Cardiology/Gastroenterology and the American Heart Association recommend prophylactic treatment with a PPI in those patients who require DAPT and those patients who are at high risk of gastrointestinal injury [1], recent studies have shown clopidogrel and PPIs to be metabolized by the same cytochrome P450 2C19 (CYP2C19) pathway [2].

Several studies showed no interaction between clopidogrel and PPIs. For example, Rassen et al. showed a slight increase in the rate of Myocardial Infarction (MI) and mortality in older patients discharged on clopidogrel and PPIs, but the authors were not able to conclude any interaction between PPIs and clopidogrel in terms of major clinical relevance [3]. Zairis et al. also showed no impact of omeprazole on the clinical efficacy of clopidogrel during the first year following PCI [4].

However, decrease in antiplatelet effects of clopidogrel with the concomitant use of PPIs has been observed. Patients had a higher level of platelet reactivity which resulted in an increased risk of adverse clinical outcomes [5]. For example, Gupta et al. concluded that the concomitant use of clopidogrel with PPIs following coronary stents implantation was associated with a significantly higher risk of major adverse cardiac events (MACEs) [6].

In 2012, Huang et al. conducted a meta-analysis based on the current idea, using old data (2009–2011) [7]. Results from their meta-analysis showed significantly increased risk of MACEs in patients with the concomitant use of clopidogrel and PPIs. Unfortunately, the high level of heterogeneity observed among the different subgroups analyzed was their major limitation.

Recently, many new studies were published based on the cardiovascular outcomes observed in patients treated with clopidogrel plus PPIs and clopidogrel alone following PCI. However, whether these controversies still exist in this new era is not clear. Therefore, we aim to show if the concomitant use of clopidogrel and PPIs is still associated with higher adverse outcomes following PCI using data obtained from recently published studies (2012 to 2016).

## Methods

### Data sources and search strategy

Three reviewers (P.K.B, A.R.T and A.B) carefully searched EMBASE, PubMed/Medline databases, and the Cochrane library for Randomized Controlled Trials (RCTs) and observational studies comparing the concomitant use clopidogrel with PPI and clopidogrel alone following PCI. The terms ‘proton pump inhibitor and clopidogrel’, ‘proton pump inhibitor and percutaneous coronary intervention’ and ‘proton pump inhibitor and dual antiplatelet therapy’ were searched carefully. In addition, abbreviations such as PPI, PCI and DAPT were also used. In order to widen the search process, individual PPIs namely ‘omeprazole, pantoprazole, lansoprazole, esomeprazole, and rabeprazole’ were also used in this search strategy. Because this current meta-analysis was based on recently published English articles, and since the previously published meta-analysis already included old data published before or in the year 2011, only studies published after the year 2011 (2012 to 2016) were considered relevant. Unpublished data were not included.

### Inclusion and exclusion criteria

RCTs and observational studies were included if:

- They compared patients treated with (clopidogrel and PPIs) and patients treated with clopidogrel but without PPIs following coronary stenting.
- Adverse cardiovascular outcomes were reported as their clinical endpoints.
- They were published after the year 2011.

RCTs and observational studies were excluded if:

- They did not compare patients (clopidogrel and PPIs) with clopidogrel alone following coronary stenting.
- Adverse cardiovascular outcomes were not reported as their clinical endpoints.
- They were published before or in the year 2011.
- They were duplicates.

### Outcomes and follow up periods

Reported outcomes which have been listed in Table 1 included:

**Table 1:** Reported outcomes and their follow up periods

Study	Reported outcomes	Follow up periods	Type of follow up
Bhurke 2012 [17]	MI and revascularization	9 months	Short term
Burkard 2012 [18]	Death, MI, ST, MACE, TVR	3 years	Long term
Chitose 2012 [19]	Death, MI	18 months	Long term
Douglas 2012 [20]	Death, MI	10 months	Short term
Dunn 2013 [21]	Death, MI, TVR	1 month	Short term
Goodman 2012 [22]	Death, MI, ST	1 year	Long term
Hsieh 2015 [23]	MI, revascularization	1 year	Long term
Macaione 2012 [24]	Death, MI, TVR	3 years	Long term
Weisz 2015 [25]	Death, MACEs, MI, ST, TVR	In hospital, 2 years	Short and long term
Zou 2014 [26]	Death, MACEs, MI, ST, TVR	1 year	Long term
Gargiolo 2016 [16]	Death, MACEs, MI, ST	2 years	Long term

*Abbreviations*: *MI* Myocardial infarction, *ST* Stent thrombosis, *MACEs* Major adverse cardiac events, *TVR* Target vessel revascularization

- All-cause mortality
- MI
- Target vessel revascularization (TVR)
- Stent thrombosis (ST)
- MACEs which consisted of death, MI and repeated revascularization.

Follow up period was divided into a short term follow up period (<1 year) and a long term follow up period (≥ 1 year).

### Data extraction and quality assessment

Three authors (P.K.B, A.R.T and A.B) independently reviewed the data extracted from the studies included in this meta-analysis. Information regarding the type of study, the total number of patients in the study group and the control group respectively, data regarding the baseline characteristics of the patients involved, information regarding the cardiovascular outcomes reported as well as the follow up periods associated with each eligible study were systematically extracted. At a certain point, when the authors disagreed about including certain studies, disagreements were resolved and a final decision was made by the fourth author (W.Q.H). Since only two trials were included in this meta-analysis whereas the other studies were observational cohorts, the risk of bias was not assessed [8].

### Methodological quality and statistical analysis

Recommendations from the Preferred Reporting Items for Systematic Reviews and Meta-Analyses guideline were followed [9]. Heterogeneity was assessed using the following:

- Cochrane Q-statistic test based on a *P* value with a cut-off point of 0.05 whereby a value less or equal to 0.05 was considered statistically significant.
- I^2^-statistic test whereby an increasing value denoted an increasing heterogeneity.

A fixed effects model (I^2^ < 50%) or a random effects model (I^2^ > 50%) was used based on the value of I^2^ obtained.

Odds Ratios (OR) with 95% Confidence Intervals (CIs) were calculated. The pooled analyses were performed with RevMan 5.3 software.

Publication bias was assessed by observing funnel plots. The reason for using funnel plots was the fact that studies with a smaller volume were used. For studies of smaller volumes, due to the higher degree of random changes, they have a wider distribution of results compared to studies of greater volumes. This might cause asymmetry in the funnels whereby publication bias could therefore be visually estimated.

Ethical approval was not necessary for such types of research articles.

## Results

### Study selection

**Fig. 1:**
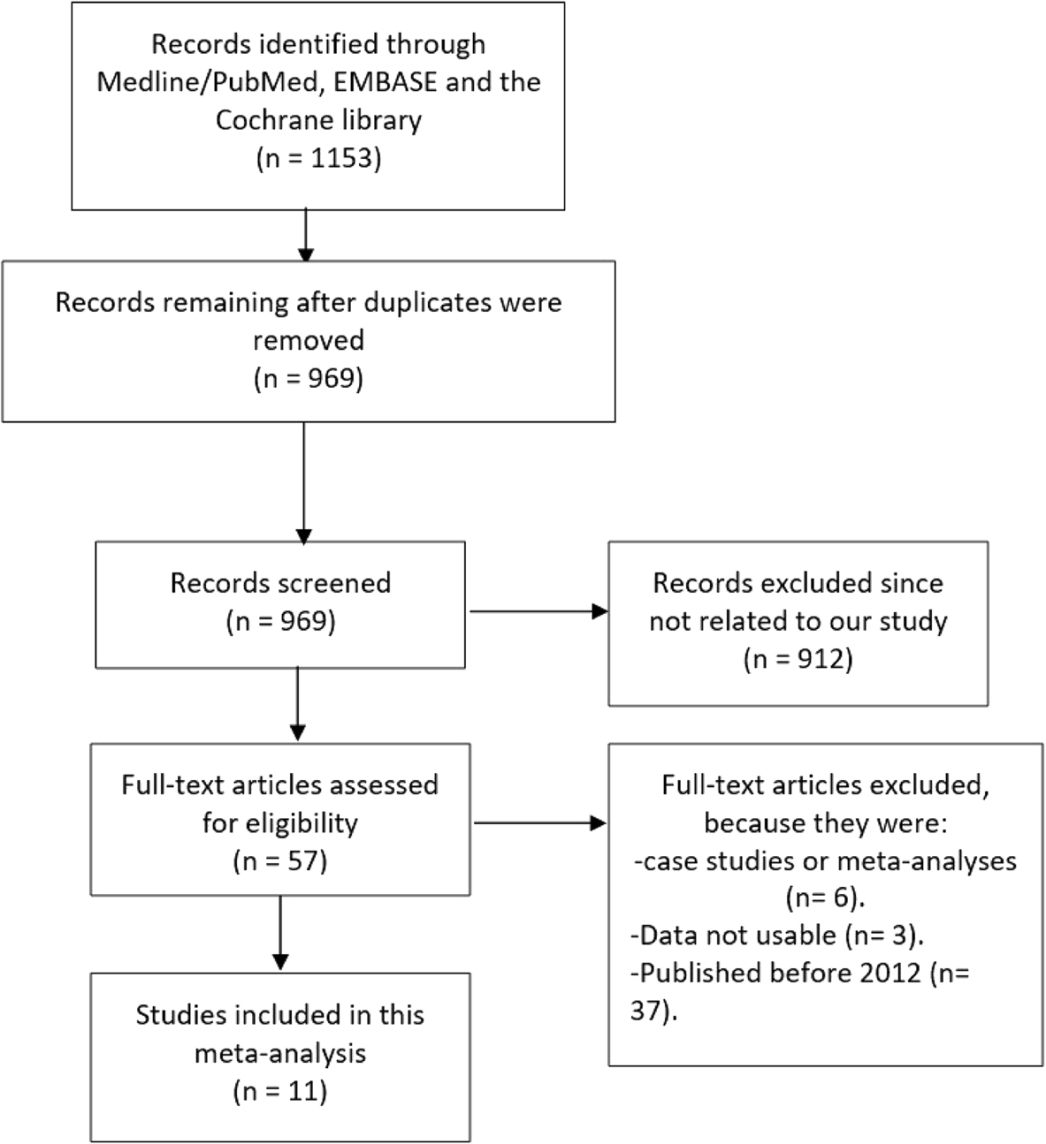
Flow diagram for the study selection

A total number of 1153 articles were obtained from the searched databases. One thousand and ninety-six articles were rejected since they were either not related to this current topic or they were duplicates. Fifty-seven full text articles were assessed for eligibility. A further six articles were eliminated since they were case studies and meta-analyses. Three more articles were eliminated because their data could not be used (outcomes were reported in terms of Hazard Ratio which was not appropriate to be used in meta-analysis). In addition, 37 more articles were eliminated since they were published before the year 2012. Finally, 11 articles were included in this analysis (Fig. 1).

### Baseline characteristics

A total number of 84,729 patients were included in this analysis (29,235 patients treated with clopidogrel plus PPIs and 55,494 patients treated with clopidogrel alone). The general features of the studies have been summarized in Table 2.

**Table 2:** General features of the studies included

Studies	No of patients using clopidogrel + PPIs (*n*)	No of patients using clopidogrel alone (*n*)	Type of study	Region
Bhurke 2012	2958	7143	Retrospective	United states
Burkard 2012	109	692	Retrospective	United states
Chitose 2012	187	443	Observational	Japan
Douglas 2012	12439	16900	Observational	United Kingdom
Dunn 2013	408	9191	Retrospective	Charlottesville
Goodman 2012	3255	6021	RCT	Canada
Hsieh 2015	670	5933	Observational	Taiwan
Macaione 2012	121	55	Retrospective	Italy
Weisz 2015	2162	6419	Observational	Israel
Zou 2014	6188	1465	Observational	China
Gargiolo 2016	738	1232	RCT	Italy
Total no of patients (*n*)	29,235	55,494		

*Abbreviations*: *PPIs* Proton pump inhibitor, *RCT* Randomized controlled trial

Study Douglas 2012, which was conducted in United Kingdom, consisted of the highest number of patients, followed by the studies Bhurke 2012, Dunn 2013 and Goodman 2012 respectively.

The baseline features of the patients have been listed in Tables 3 and 4 lists the different types of PPIs used by the patients.

**Table 3:** Baseline characteristics of the patients

Study	Age (year)	Males (%)	HT (%)	Ds (%)	DM (%)	CS (%)
	C + PPI/C alone	C + PPI/C alone	C + PPI/C alone	C + PPI/C alone	C + PPI/C alone	C + PPI/C alone
Bhurke 2012	61.5/59.6	68.7/76.3	-	-	28.9/26.4	-
Burkard 2012	66.5/63.3	68.8/79.9	72.5/65.0	73.4/75.9	29.6/17.2	24.8/29.8
Chitose 2012	70.3/68.9	71.6/72.2	77.9/79.0	61.9/61.7	35.3/33.7	23.9/26.2
Douglas 2012	71.0/68.0	58.0/65.0	-	-	34.0/29.0	16.0/18.0
Dunn 2013	63.9/62.5	69.3/72.3	49.5/51.5	39.4/41.2	16.5/20.3	23.9/29.6
Goodman 2012	63.0/62.0	72.4/71.2	65.6/65.4	49.8/45.0	25.8/24.7	36.2/35.7
Hsieh 2015	68.4/66.5	63.6/66.4	-	-	-	-
Macaione 2012	63.7/65.8	80.2/87.3	70.2/81.8	53.7/58.2	41.3/49.1	37.2/27.3
Weisz 2015	64.4/63.2	70.1/75.9	83.7/77.8	76.9/73.2	34.8/31.4	22.7/22.6
Zou 2014	66.2/65.7	73.5/73.9	71.3/70.4	60.2/62.3	25.8/23.6	32.2/31.0
Gargiolo 2016	71.2/68.1	72.5/79.2	72.5/71.3	53.8/55.3	23.3/24.8	22.6/24.4

*Abbreviations*: *C* Clopidogrel, *PPI* Proton pump inhibitor, *HT* Hypertension, *Ds* Dyslipidemia, *DM* Diabetes mellitus, *CS* Current smoker

**Table 4:** Types of Proton Pump Inhibitors used by the patients

Studies	Omeprazole	Esomeprazole	Lansoprazole	Pantoprazole	Rabeprazole
Bhurke 2012	27.1	23.1	17.6	25.8	6.30
Burkard 2012	17.0	51.0	7.00	25.0	-
Chitose 2012	-	-	-	-	-
Douglas 2012	-	-	-	-	-
Dunn 2013	14.0	-	18.1	3.03	1.60
Goodman 2012	48.9	11.7	7.80	30.1	1.48
Hsieh 2015	-	-	-	-	-
Macaione 2012	43.0	11.6	10.7	34.7	-
Weisz 2015	-	-	-	-	-
Zou 2014	-	-	-	-	-
Gargiolo 2016	0.5	0.5	90.9	7.60	0.5

Percentage (%) has been used to represent these data

According to the baseline features, there was no significant difference among the patients who were treated with (clopidogrel plus PPIs) and clopidogrel alone.

### Main results of this meta-analysis

**Fig. 2:**
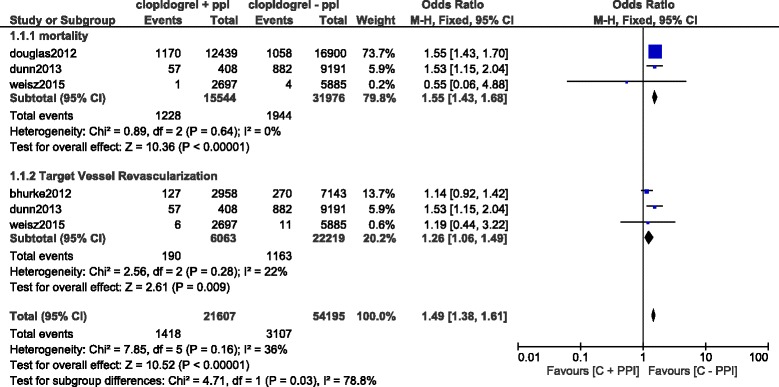
Short term adverse clinical outcomes associated with the concomitant use of clopidogrel and PPIs

**Fig. 3:**
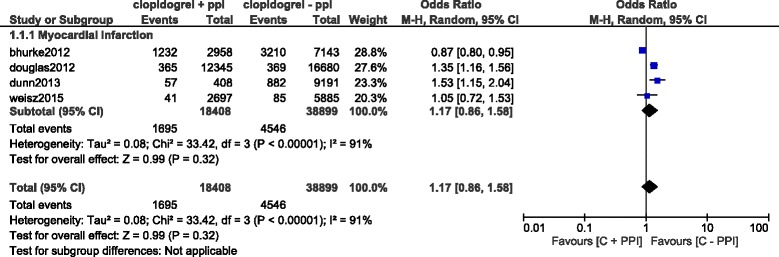
Short term Myocardial Infarction associated with the concomitant use of clopidogrel and PPIs

Results of this analysis (summarized in Table 5) showed that during a short term follow up period, using a fixed effects model, mortality and TVR significantly favored clopidogrel alone with OR: 1.55; 95% CI: 1.43–1.68, *P* < 0.00001 and OR: 1.26; 95% CI: 1.06–1.49, *P* = 0.009 respectively. This result has been represented in Fig. 2. However, result for the short-term MI which was analyzed using a random effects model, was not statistically significant with OR: 1.17; 95% CI: 0.86–1.58, *P* = 0.32 (Fig. 3).

**Table 5:** Results of the main analysis

Outcomes analyzed	OR with 95% CI	*P* value	I^2^ (%)
Short term follow up			
Mortality	1.55 [1.43–1.68]	0.00001	0
TVR	1.26 [1.06–1.49]	0.009	22
MI	1.17 [0.86–1.58]	0.32	91
Long term follow up			
MACEs	1.37 [1.23–1.53]	0.00001	0
MI	1.41 [1.26–1.57]	0.00001	29
ST	1.38 [1.13–1.70]	0.002	0
Mortality	1.26 [0.99–1.60]	0.06	61
TVR	1.28 [1.01–1.61]	0.04	72

*Abbreviations*: *OR* Odds ratio, *CI* Confidence interval, *TVR* Target vessel revascularization, *MI* Myocardial infarction, *MACEs* Major adverse cardiac events, *ST* Stent thrombosis

**Fig. 4:**
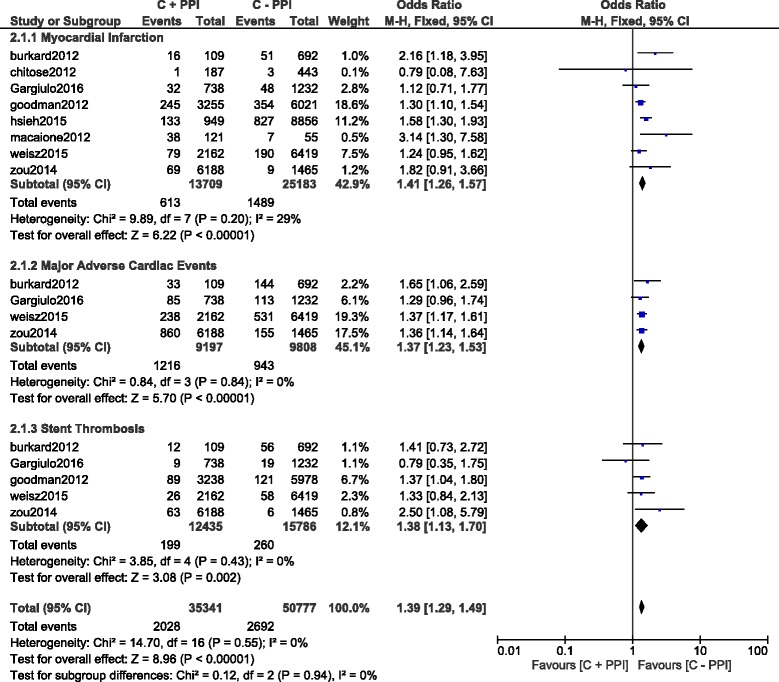
Long term adverse clinical outcomes associated with the concomitant use of clopidogrel and PPIs

During the long-term follow up period, MACEs, MI and ST significantly favored clopidogrel alone with OR: 1.37; 95% CI: 1.23–1.53, *P* < 0.00001, OR: 1.41; 95% CI: 1.26–1.57, *P* < 0.00001 and OR: 1.38; 95% CI: 1.13–1.70, *P* = 0.002 respectively (Fig. 4).

**Fig. 5:**
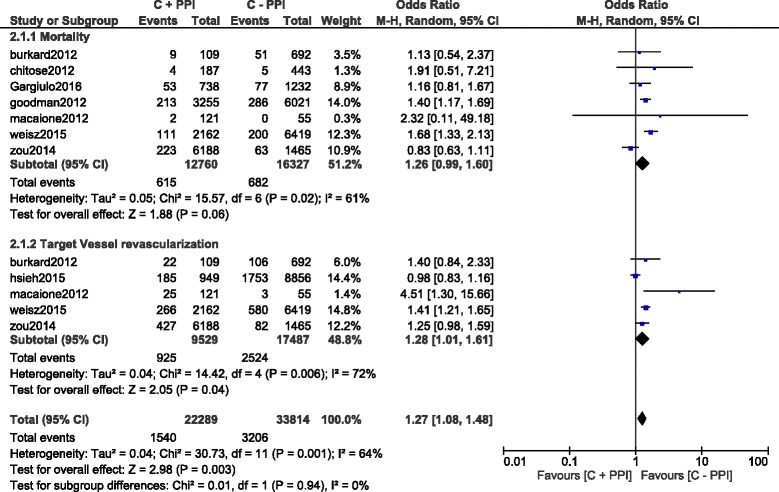
Long term mortality and TVR associated with the concomitant use of clopidogrel and PPIs

However, since a high level of heterogeneity was observed when analyzing the long-term mortality and TVR, a random effects model was used. Long term TVR also significantly favored the non-PPI group with OR: 1.28; 95% CI: 1.01–1.61, *P* = 0.04 whereas the result for the long-term mortality was not statistically significant with OR: 1.26; 95% CI: 0.99–1.60, *P* = 0.06 (Fig. 5).

**Fig. 6:**
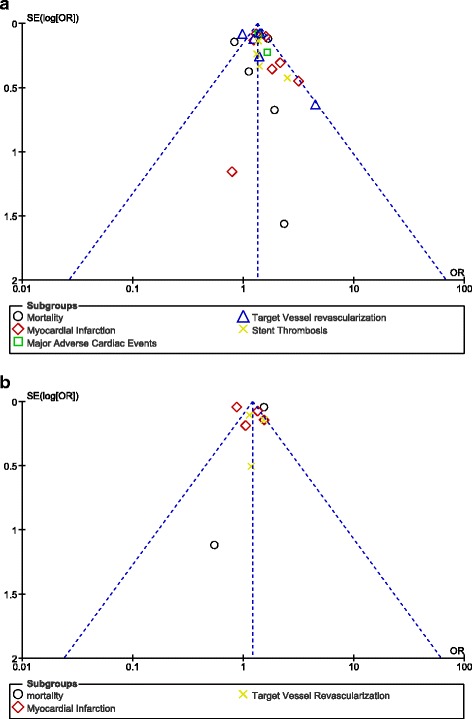
**a** and **b** Funnel plots showing publication bias

Based on a visual inspection of the funnel plot, there has been a low evidence of publication bias among the studies that assessed several subgroups of adverse cardiovascular endpoints. These funnel plots have been illustrated in Fig. 6a and b.

## Discussion

Controversies still exist with the concomitant use of clopidogrel and PPIs following coronary stenting, which remain to be solved in this new era. In this analysis, we aimed to compare the adverse clinical outcomes associated with the concomitant use of clopidogrel and PPIs versus clopidogrel alone following PCI using data obtained from recently published articles (2012–2016).

This current analysis showed that during a short term follow up period, mortality and revascularization were significantly lower in those patients who did not require treatment with PPIs. Moreover, during the long term follow up period, adverse cardiovascular outcomes such as MACEs, ST, MI and TVR significantly favored patients in the non-PPI group. However, result for the long-term mortality was similar manifested in both groups.

The previously published meta-analysis [7] which included 32 studies with publication date before the year 2012 (29 studies published in English and 3 studies published in Chinese), showed the concomitant use of PPI and clopidogrel to be associated with higher MACEs with OR: 1.27, 95% CI: 1.13–1.42 when a combination of data obtained from randomized trials and observational studies was used. However, pooling data only from randomized trials did not show any increase risk of MACEs with OR: 0.92, 95% CI: 0.53–1.58; *P* = 0.72, I^2^ = 0%. When mortality was analyzed using a random effects model, a significant increase was observed with HR: 1.30, 95% CI: 0.91–1.86. But when a fixed effects model was used to analyze mortality, no significant increase was observed with clopidogrel plus PPI with OR: 0.92, 95% CI: 0.82–1.04.

Several reasons have been suggested for such a result. First of all, PPIs involve the same metabolic pathway (mainly CYP2C19 isoenzyme) with that of clopidogrel [10]. In other words, by occupying the same metabolic pathway as clopidogrel, PPIs are expected to reduce the antiplatelet effects of clopidogrel. Because PPIs can act as both, inhibitors and substrates of CYP2C19, patients treated with clopidogrel and PPIs are vulnerable to a reduced effectiveness of clopidogrel. This could in turn result in a higher platelet activity following PCI finally causing an increase in adverse clinical outcomes. Gilard et al. were the first ones to show the interaction of clopidogrel and PPIs [11]. Moreover, PPIs not only showed a high platelet reactivity but also showed an increased inflammatory state due to the rise in the level of interleukins-6 which in turn could increase the occurrence of ischemic events [12]. However, whether PPIs really have an effect on clopidogrel’s antiplatelet effect is still being debated.

Similar to the results of this current analysis, many other previously published studies showed that adverse clinical outcomes were significantly increased in the PPIs group. Gupta et al. concluded that the concomitant use of clopidogrel with PPIs was associated with an increased risk of MACEs following PCI [6]. In addition, Ho et al. showed increased risk of adverse outcomes with clopidogrel plus PPIs [13].

However, even if many studies supported these current results, several other studies showed results which were completely different. For example, Rassen et al. showed that although a slight increase in hospitalization due to MI and death was observed in older patients who were prescribed PPIs and clopidogrel together, there was not enough evidence to conclude any major interaction between these 2 drugs [3]. In the analysis from the Guthrie Health Off-Label Stent (GHOST) Investigators, the authors also concluded that the use of PPIs with DAPT was not associated with any increase in MACEs following PCI [14]. However, their study had a follow up period of only 6 months. Zairis et al. also showed no impact of omeprazole on the clinical efficacy of clopidogrel during the first year following successful PCI [4]. However, the authors concluded that further highly powered studies should be conducted to confirm whether or not, omeprazole has any effect on the antiplatelet mechanism of clopidogrel. In addition, the COGENT study also did not observe any apparent interaction between clopidogrel and omeprazole, but however, the authors strictly mentioned that their results did not rule out clinically meaningful differences in adverse cardiovascular outcomes due to the use of PPIs [15].

Nevertheless, among all the PPIs, omeprazole is considered to have a higher effect on the mechanism of clopidogrel. Other studies did not show any notable inter-reaction among non-omeprazole PPIs and clopidogrel. For example, when pantoprazole was used along with clopidogrel, no increase in adverse events was observed and therefore pantoprazole has been recommended compared to omeprazole in patients treated with clopidogrel. In addition, in a sub-analysis of the randomized PRODIGY trial, it was reported that the concomitant use of PPIs, when clinically indicated, in patients receiving clopidogrel, was not associated with adverse clinical outcomes. However, it should also be noted that only less than 1.5% of the patients used omeprazole while more than 90% of the patients in that particular trial used lansoprazole, suggesting that this type of PPIs might be safer to use with clopidogrel [16].

Since the adverse clinical events associated with non-omeprazole PPIs and clopidogrel have still not clearly been studied, further research is recommended with these individual PPIs (esomeprazole, rabeprazole, lansoprazole, and pantoprazole). In addition, bleeding events especially gastrointestinal bleeding associated with the concomitant use of clopidogrel with these individual PPIs should also be carefully studied.

A moderate level of heterogeneity was observed among certain subgroups analyzing the cardiovascular outcomes. Only English publications were considered, and articles written in other languages were ignored, therefore, a language bias might most probably be present. Moreover, data obtained from conference abstracts and other unpublished studies were not included. However, since most of the data used in this analysis were obtained from observational studies, it could be one of the reasons contributing to the moderate risk of bias observed. In addition, a high level of heterogeneity could also have been due to the fact that different types of patients were included (chronic stable angina, STEMI, NSTEMI) and the type of stent following PCI was also not taken into consideration; patients implanted with DES and BMS were combined and analyzed.

Novelty in this study is the fact that a lower level of heterogeneity was present among several subgroups compared to the previously published meta-analysis. Moreover, different from other studies which mainly report either short term, mid-term or long term outcomes, this analysis has compared the long term and short term adverse clinical outcomes in patients with and without the concomitant use of clopidogrel and PPIs. In addition, this analysis included data obtained from newly published research articles.

### Limitations

Several limitations are present. Due to a limited number of patients, the result of this analysis might be affected. Moreover, this analysis involved mainly data obtained from observational studies which might be another limitation, and because of this reason, the bias risk of the studies included in this analysis was not assessed using recommendations from the Cochrane Collaboration. In addition, one study reported death, MI and revascularization together. Since data for each outcome could not be separated, we have included the same data for this particular study in the different subgroups analyzing mortality, MI and TVR. Also, adverse bleeding events (GI bleeding) were not analyzed because only a few studies reported bleeding outcomes, which were also different in each of the study, making it difficult to compare.

## Conclusion

The combined use of clopidogrel with PPIs is still associated with significantly higher adverse cardiovascular events such as MACEs, ST and MI following PCI supporting results of the previously published meta-analysis. However, long-term mortality is not statistically significant warranting further analysis with randomized patients.

## Abbreviations

DAPT: Dual antiplatelet therapy
MACEs: Major adverse cardiac events
PCI: Percutaneous coronary intervention
PPI: Proton pump inhibitor
ST: Stent thrombosis
